# Q Fever Visceral Abscesses After Contact With Native Australian Marsupials

**DOI:** 10.5694/mja2.70305

**Published:** 2026-09-22

**Authors:** Samantha J. Laws, Enzo Binotto, James Stewart, Jennifer Ho, Trent Yarwood, Josh Hanson, Simon Smith

**Affiliations:** ^1^ Cairns Hospital Cairns Queensland Australia; ^2^ James Cook University Cairns Queensland Australia; ^3^ The Kirby Institute Sydney New South Wales Australia

**Keywords:** Q fever, vaccine preventable disease, zoonoses

## Abstract

We describe two patients in Far North Queensland with acute Q fever. One patient had their infection complicated by a splenic abscess, the other developed a hepatic abscess. Both patients had contact with small Australian marsupials in the weeks before presentation and both required prolonged doxycycline to treat their infection.

## 1 Clinical Record 1

A 64‐year‐old man living in metropolitan Cairns presented to hospital with 2 days of confusion. He was a smoker but was otherwise well, took no regular medications and worked as a crane operator. He recalled burying a dead bandicoot he found in his garden 2 weeks before presentation where he wore gloves but no face mask. On admission he was febrile (38.7°C), hypotensive (blood pressure 88/50 mmHg) and had hypoxic respiratory failure (oxygen saturation 92% on oxygen by high‐flow nasal prongs, with a fraction of inspired oxygen [FiO_2_] of 0.45 delivered at 40 L/min). Blood tests revealed thrombocytopenia (51 × 10^9^/L; reference interval [RI], 140–400 × 10^9^/L), acute kidney injury (creatinine, 246 μmol/L; RI, 36–73 μmol/L) and elevated liver enzymes (total bilirubin, 36 μmol/L [RI, < 20 μmol/L]; alanine aminotransferase [ALT], 101 U/L [RI, < 45 U/L]; aspartate aminotransferase [AST], 337 U/L [RI, < 35 U/L]).

**FIGURE 1:**
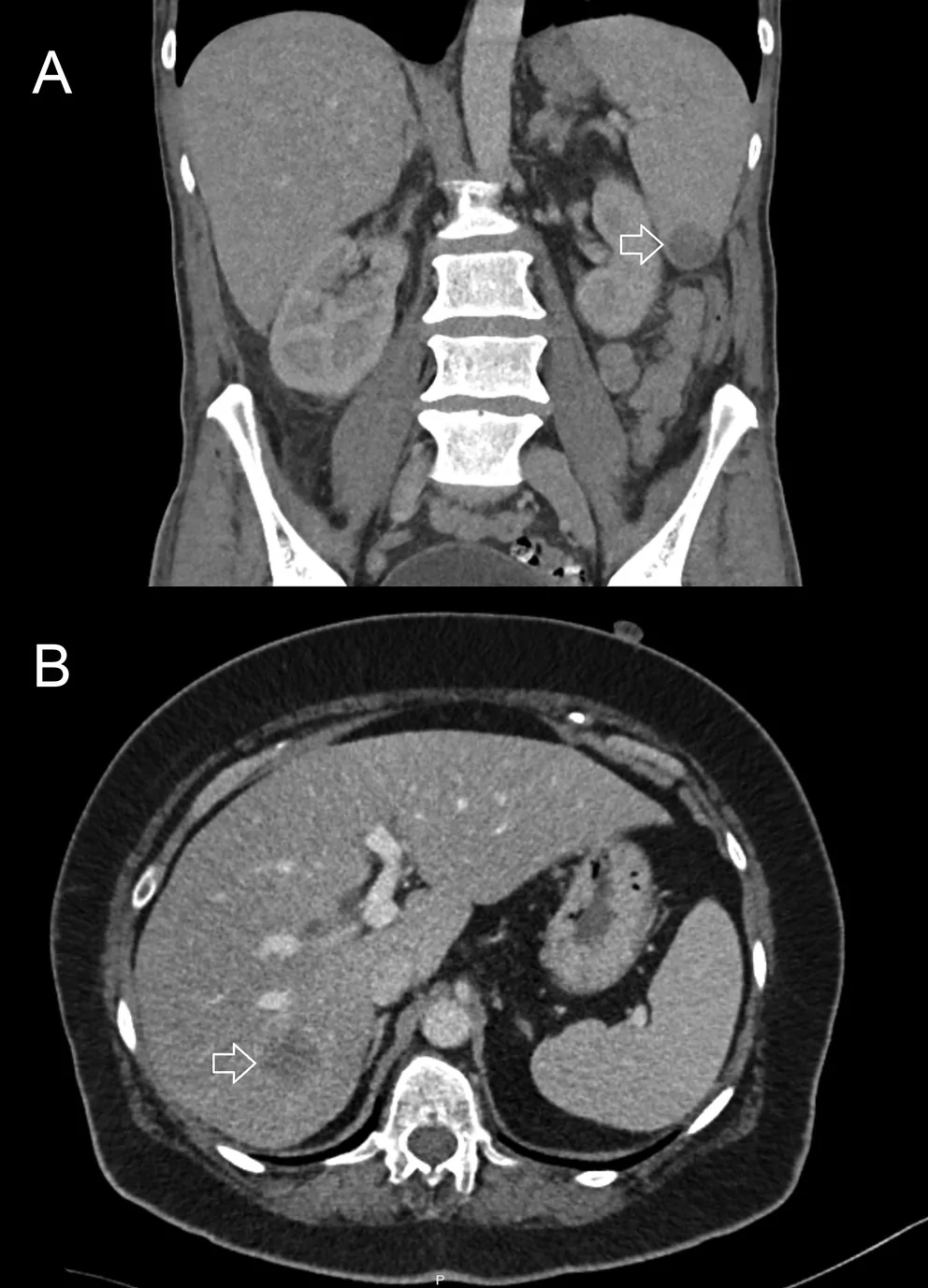
(A) Coronal computed tomography (CT) images show a splenic abscess (arrow). (B) Axial CT images reveal a liver abscess (arrow). Both lesions were aspirated and Q fever polymerase chain reaction testing of the fluid was positive.

Computed tomography (CT) demonstrated bilateral bronchopneumonic changes, hepatomegaly and splenomegaly with a 3.9 cm lesion in the inferior pole. He was diagnosed with severe community‐acquired pneumonia, commenced on intravenous meropenem and azithromycin and admitted to the intensive care unit for vasopressor support. Q fever polymerase chain reaction (PCR) on blood collected on Day 2 of his admission was positive and Q fever Phase 2 immunoglobulin M (IgM) was reactive. His antibiotics were changed to doxycycline 100 mg twice daily. Repeat CT imaging revealed ongoing multilobar pneumonia and a splenic abscess (Figure 1A). Q fever PCR was positive in both sputum and fluid obtained from CT‐guided aspiration of his splenic abscess. He continued doxycycline and, following clinical improvement and resolution of the splenic abscess on repeat imaging, he was able to cease after 3 months of therapy.

## 2 Clinical Record 2

A 69‐year‐old woman from rural Far North Queensland presented to her local hospital with 5 days of fevers, fatigue, myalgia and nausea. She had a history of Type 2 diabetes mellitus, hypertension and asthma. Her symptoms were attributed to a respiratory viral infection, and she was discharged home but represented 3 days later with continuing fevers. She recalled a quoll exposure 1 week before becoming unwell, having discovered the animal trapped in her car and had subsequently cleaned scat from the seats. There was no other history of animal exposure.

Physical examination revealed cervical lymphadenopathy. She was thrombocytopenic (134 × 10^9^/L; RI, 140–400 × 10^9^/L), with an acute kidney injury (creatinine, 84 μmol/L; RI, 36–73 μmol/L) and had elevated liver enzymes (γ‐glutamyl transferase [GGT], 89 U/L [RI, < 38 U/L]; ALT, 117 U/L [RI, < 34 U/L]; AST, 81 U/L [RI, < 31 U/L]). Blood Q fever PCR on Day 1 of admission was positive, but serology was non‐reactive. She commenced oral doxycycline 100 mg twice daily and was discharged home. She re‐presented to hospital 3 days later with ongoing fevers and right upper quadrant tenderness. An abdominal CT scan revealed a liver abscess (Figure 1B). She was transferred to Cairns Hospital, where an ultrasound‐guided aspiration of the abscess was Q fever‐positive on PCR testing. She continued doxycycline for 4 weeks and repeat Q fever Phase 2 IgM and immunoglobulin G (IgG) collected at the end of treatment were both reactive.

## 3 Discussion

**FIGURE 2:**
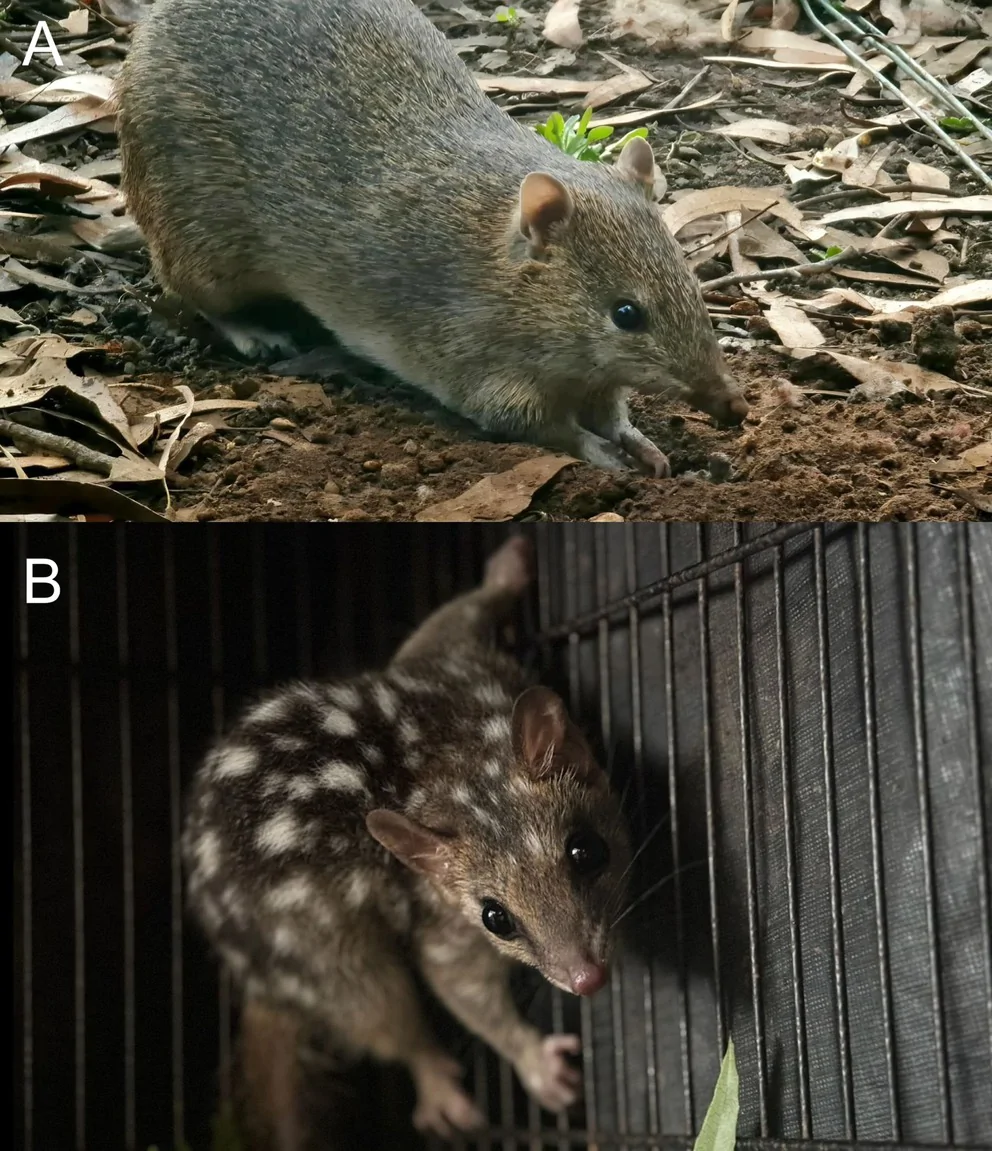
Images taken by a volunteer from a local wildlife rescue group of (A) a northern brown bandicoot and (B) a northern quoll.

Q fever is a zoonotic infection caused by the intracellular bacterium *Coxiella burnetii* . Humans develop Q fever through direct or indirect contact with animals that excrete the organism into the environment. Globally, cattle, sheep and goats are the most common reservoirs for Q fever, but, in Australia, feral mammals and native Australian marsupials, including kangaroos, koalas, possums, bandicoots (Figure 2A) and quolls (Figure 2B), may also be responsible [1, 2, 3]. Q fever has been detected in marsupials in multiple Australian states, including Queensland, New South Wales and Western Australia, with most human cases occurring in distinct geographical regions of Queensland and New South Wales [4]. Neither of our patients had contact with farms, stockyards, abattoirs or other animals, making exposure to the marsupials the likely source of their infection.

The clinical manifestations of acute Q fever vary, but symptoms often include fever, headache, myalgia and fatigue. Q fever is usually a mild illness but can be life‐threatening [5]. Pneumonia and hepatitis are common but acute visceral abscesses are rare [6]. It is estimated that about 5% of acute Q fever cases develop chronic infection, which can include endocarditis, osteomyelitis, endovascular infection and granulomatous hepatitis [7, 8].

Acute Q fever is diagnosed with serology and molecular testing. Where available, PCR testing can be performed on blood and fluid and/or tissue and is useful in the early acute setting before serology is diagnostic. In our patients, PCR was performed in a National Association of Testing Authorities (NATA)‐accredited laboratory enrolled in a Q fever quality assurance program. Serology is commonly repeated at 3, 6 and 12 months, along with assessment of symptoms, to assess for the development of chronic Q fever [9].

The management of Q fever is incompletely defined; 2 weeks of oral doxycycline is usually sufficient to treat acute infection, but chronic Q fever often requires combination antimicrobial therapy for up to 24 months [10]. Vaccination against Q fever is available but requires pre‐vaccination testing and is currently only recommended for individuals at highest risk, including abattoir workers, veterinarians, wildlife and zoo workers [11]. Both our cases had incidental exposure to host animals and, therefore, would not currently have been considered for vaccination.

## 4 Lessons From Practice

- Reservoirs for Q fever in Australia include domestic ruminants, feral mammals and native marsupials.
- Q fever should be considered as a differential in the workup of visceral abscesses, particularly in people with a history of exposure to these animals.
- Q fever polymerase chain reaction testing can be performed on blood, tissue and abscess fluid, which may expedite diagnosis and the initiation of appropriate antimicrobial therapy.
- The optimal duration of antibiotic therapy for Q fever hepatic and splenic abscesses is poorly defined, but patients may require several weeks or months of treatment.

## Author Contributions

**Samantha J. Laws:** conceptualisation; duration curation; methodology; visualisation; writing (original draft). **Enzo Binotto:** writing (review and editing). **James Stewart:** writing (review and editing). **Jennifer Ho:** writing (review and editing). **Trent Yarwood:** writing (review and editing). **Josh Hanson:** writing (review and editing). **Simon Smith:** conceptualisation; methodology; visualisation; supervision; writing (review and editing).

## Funding

The authors have nothing to report.

## Disclosure

Not commissioned; externally peer reviewed.

## Consent

Both patients included in this manuscript provided written informed consent for publication of personal material in the *Medical Journal of Australia*.

## Conflicts of Interest

The authors declare no conflicts of interest.

## Data Availability

This article includes no original data.
